# A Review on the Use of Metformin in Pregnancy and Its Associated Fetal Outcomes

**DOI:** 10.7759/cureus.30039

**Published:** 2022-10-07

**Authors:** Vaishnavi Verma, Ashok M Mehendale

**Affiliations:** 1 Obstetrics/Gynaecology, Jawaharlal Nehru Medical College, Wardha, Datta Meghe Institute of Medical Sciences (DMIMSU), Wardha, IND; 2 Preventive Medicine, Datta Meghe Institute of Medical Sciences, Wardha, IND

**Keywords:** gestational diabetes mellitus, polycystic ovary syndrome, maternal obesity, fetal outcomes, small for gestational age, metformin, pre-eclampsia

## Abstract

A commonly used first-line anti-diabetic medication, metformin, has been used in pregnancy. The drug is known to have specific effects on different organs around the body. One of these organs includes the ovaries. Therefore, for more than 40 years, it has often been prescribed for maternal obesity along with gestational diabetes mellitus. Untreated pregnancies like these frequently result in complications for both the mother and the fetus, like macrosomia, pregnancy-induced hypertension, obstructed labor, stillbirths, and perinatal deaths. In addition, there is also evidence that these mothers tend to develop type II diabetes mellitus during their pregnancy and even a few years post-delivery. These complications can be controlled or even reduced with the help of metformin, sometimes combining it with insulin or clomiphene citrate if required. There is still a need to cautiously prescribe the drug by outweighing its benefits against the risk associated with it. The current research on the subject leans more towards the benefits offered to the mother during pregnancy. Only a few randomized, controlled trials have been conducted on the fetal condition after the mother has been administered metformin.

Furthermore, these studies lack the appropriate sample size and long-term follow-up on these metformin-exposed offspring. As a result, there are no reliable data available to clinicians and physicians about the drug. Owing to its benefits in certain pregnancies, it is less likely that the drug will cease to be prescribed. Therefore, it becomes increasingly imperative to conduct more research on this topic to ensure the drug is safe for the mother and the offspring.

## Introduction and background

Diabetes, especially type II/adult/non-insulin-dependent diabetes mellitus, is the primary condition for which the oral anti-diabetic medication metformin (a biguanide derivative) is prescribed [1]. The first piece of evidence indicating its use is seen in books on herbs from the 1600s, which stated its origin in the French lilac plant [2]. In modern times, the drug was first explicitly synthesized as a dimethyl biguanide compound in 1922, around the same time synthetic insulin started being produced on the market [3]. The drug was named Glucophage, or "glucose-eater," by French scientist Jean Stearne in the 1950s due to the mechanism by which it achieves glycemic control when used for diabetes mellitus. The sheer popularity of its use can be attributed to its ease of administration (daily oral dosage as the medication is available as extended-release preparations), mild side effects, affordability, and minimal weight gain [4].

**Figure 1:**
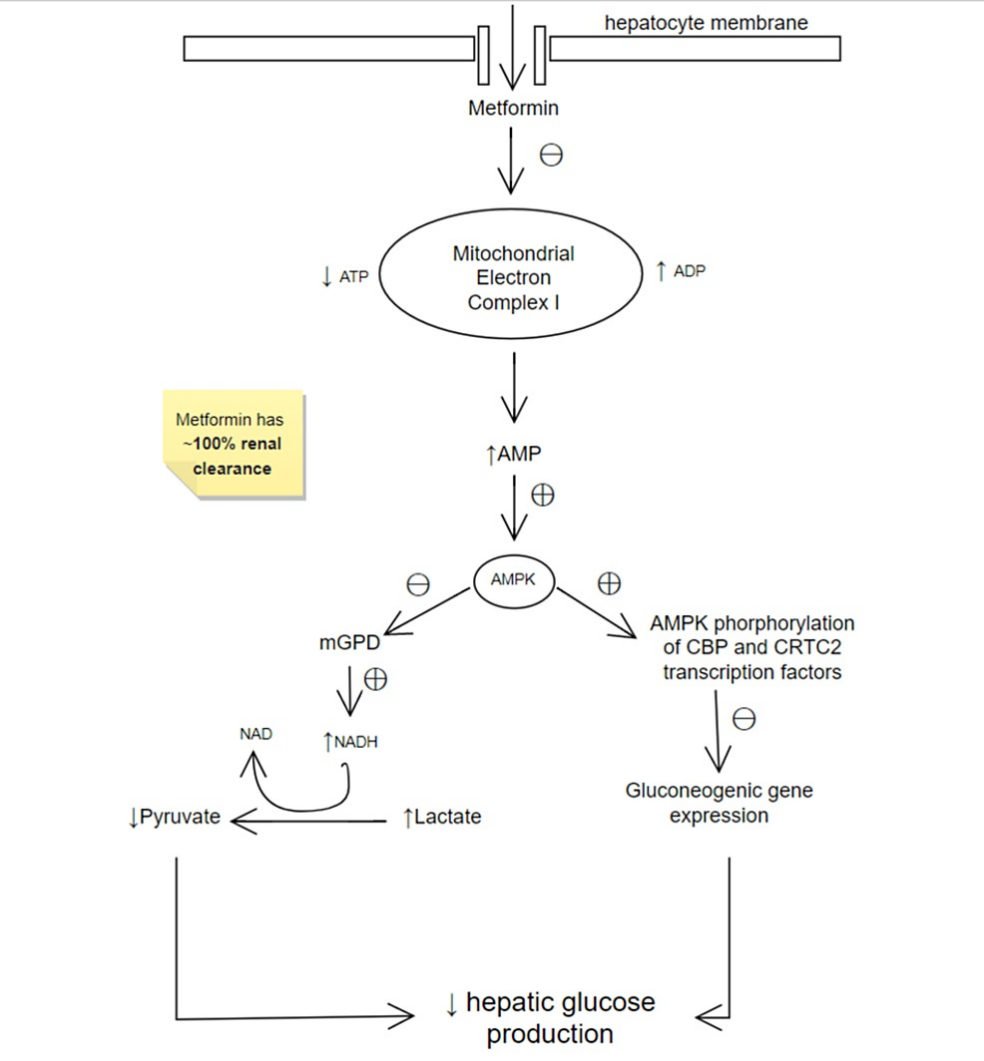
Metformin: its mechanism of action Image Credits: Vaishnavi Verma The abbreviations in the given figure are ATP: adenosine triphosphate, ADP: adenosine diphosphate, AMP: adenosine monophosphate, AMPK: AMP-activated protein kinase, mGPD: mitochondrial glycerol-3-phosphate dehydrogenase, NADH: nicotinamide adenine dinucleotide hydrogen (reduced), NAD: nicotinamide adenine dinucleotide, CBP: CREB-binding protein, and CRTC2: CREB-regulated transcription coactivator 2 (where CREB is cyclic AMP-response element binding protein).

Even though metformin has been a reliable drug prescribed for diabetes mellitus for the past few decades, its exact mode of action still remains unclear. Recent studies suggest that the drug enters the cells via transporters on their cell membranes. Therefore, alterations in these transporters lead to different outcomes with metformin therapy in such patients [5]. Metformin enters the hepatic cells via these transporters and chiefly acts in the mitochondria. Within the mitochondria, it decreases the efficiency of the electron transport chain (ETC) when it reduces the activity of complex I. Adenosine triphosphate (ATP) production is reduced as a result of this. The drug increases adenosine monophosphate (AMP) levels in the same pathway by inhibiting AMP deaminase. The resulting AMP:ATP ratio leads to the 5'AMP-activated protein kinase (AMPK) getting activated, which then sets ahead a cascade of intracellular effects that cause the stimulation and suppression of catabolic and anabolic pathways [6]. Therefore, metformin’s anti-hyperglycemic effect is achieved by decreasing hepatic gluconeogenesis, antagonizing the hormone glucagon's action, and increasing the reuptake of glucose by skeletal muscles to a certain extent, as seen in Figure 1 [7,8].

Metformin acting on different sites of the body produces specific actions. In the liver, it improves fasting plasma glucose by decreasing gluconeogenesis, and opposing glucagon signaling enhances insulin sensitivity in the muscles by boosting the number and activity of insulin receptors and glucose absorption. In the gastrointestinal tract, it increases postprandial blood glucose by decreasing intestinal glucose uptake and increasing GLP-1 secretion. It enhances insulin sensitivity in the adipose tissues while reducing inflammation. Finally, in the ovary, it may restore ovulation in those living with polycystic ovary syndrome [4]. Because of these actions, while metformin is primarily used to manage diabetes mellitus (both types 1 and 2), it has also seen its use in various other conditions, including obesity, polycystic ovary syndrome, non-alcoholic fatty liver disease, and even as an adjuvant in the prevention and treatment of upper gastrointestinal cancers [9-12].

## Review

Pregnancy and the use of metformin

For pregnant women, metformin is widely recognized as a sensible, safe, and successful treatment choice, especially in polycystic ovary syndrome and gestational diabetes mellitus. It is even prescribed for non-diabetic obese pregnant women [13].

Gestational diabetes mellitus

The pregnancy issue known as gestational diabetes mellitus occurs when a pregnant woman's blood glucose levels suddenly rise on their own. As reported by the International Diabetes Foundation (IDF) [14], gestational diabetes mellitus affects around 14% of pregnancies globally. Obesity, a lack of certain micronutrients, a family history of either insulin resistance or diabetes mellitus, and an older maternal age increase the likelihood of gestational diabetes mellitus. Pregnancy complications such as gestational diabetes mellitus can potentially be fatal. It can cause these mothers to acquire non-insulin-dependent diabetes mellitus five to ten years [15] post-delivery, obstructed labor, elevated blood pressure, and big birth weight kids [16].

It is often challenging to differentiate between gestational diabetes mellitus and pre-existing diabetes as many of these mothers were not screened for diabetes before their pregnancies. Currently, two strategies have been adopted by various international guidelines which can help diagnose gestational diabetes mellitus, namely the 'one-step' approach and the 'two-step' approach, where the former method uses a 75 g oral glucose tolerance test and the latter employs a 50 g non-fasting glucose screening which is followed up with a 100 g oral glucose tolerance test for those who screened positive (the diagnostic values seen in Table 1) [17].

**Table 1:** Different guidelines for the diagnosis of gestational diabetes mellitus. Vandorsten JP, Dodson WC, Espeland MA, et al.: NIH consensus development conference: diagnosing gestational diabetes mellitus. NIH Consens State Sci Statements. 2013, 29:1–31.

Strategy	Guidelines	Fasting Blood Glucose	One-hour postprandial blood glucose	Two hours postprandial blood glucose
One-step approach	World Health Organisation	92-125 mg/dL	180 mg/dL	153-199 mg/dL
One-step approach	National Institute for Health and Care Excellence	100 mg/dL		140 mg/dL
Two-step approach	Canadian Diabetes Association	95 mg/dL	191 mg/dL	160 mg/dL

According to several international recommendations, metformin is a medication that is frequently prescribed for gestational diabetes mellitus. The Scottish Intercollegiate Guidelines Network (SIGN) says that [18] glibenclamide or metformin may be used to lower blood sugar in cases of gestational diabetes mellitus [19]. The American Diabetes Association (ADA) [20] recognizes metformin as a category B medication that should be used for gestational diabetes mellitus as a second-line medication following insulin [21]. For gestational diabetes mellitus, the National Institute for Health and Care Excellence (NICE) advises a diet and exercise regime to achieve appropriate blood glucose levels, which, if not met within a fortnight, should be followed by the recommended administration of metformin [22].

While there is no specific treatment for gestational diabetes mellitus, it is often managed using lifestyle interventions like controlled diets and exercise to improve insulin sensitivity in pregnant mothers. As far as pharmaceutical management is concerned, metformin and insulin are frequently prescribed drugs, with metformin being preferred over insulin even though it causes nausea, vomiting, and other gastrointestinal side effects [23]. Five randomized controlled trials on this subject were conducted, and the meta-analysis concluded that metformin helps lower the mother's weight gain throughout pregnancy and reduces the occurrence of pregnancy-induced hypertension (or pre-eclampsia) [24]. Compared to insulin and Glibenclamide therapies, macrosomia, or large for gestational age births, newborn hypoglycemia, and admission of the infant to neonatal intensive care units [13] are all at a decreased risk when metformin medication is used to treat gestational diabetes mellitus [25,26].

Maternal obesity

The WHO has defined obesity as the build-up of excess fat in the body. As a result, this could cause additional health problems for the affected individual. The body mass index (BMI) is a simple approach to evaluate obesity. It is determined by dividing the individual's weight in kilograms by the square of their height in meters. Being overweight means the person has a BMI of above 25 kg/m^2^, while being obese means the BMI is over 30 kg/m^2^. Obesity is a growing global health concern since 13% of adults globally are currently obese [27]. The rise in maternal obesity is one of the key factors contributing to morbidity and mortality in both mothers and neonates [28].

Gestational diabetes mellitus, pregnancy-induced hypertension or pre-eclampsia, thrombo-embolism, cesarean birth, and labor induction [29] are the most frequent maternal problems linked to maternal obesity [30-33]. When it comes to fetal complications, stillbirths and neonatal deaths are two times more likely to occur [34]. A distinct correlation exists between fetal macrosomia and obesity during pregnancy [35]. Additionally, there is a higher chance of developing birth defects in the fetus, including spina bifida among neural tube defects, cardiac defects, multiple congenital anomaly syndromes [36], and abdominal wall defects [37].

Metformin is not a routine drug to be administered to obese pregnant mothers. However, its use is linked with reduced weight gained during pregnancy and the risk of developing pregnancy-related hypertension [29]. It can even improve neonatal outcomes by decreasing NICU admission rates [38]. Metformin usage is also linked to reduced concentrations of the inflammatory proteins CRP and interleukin-6 in circulation [39]. Metformin's anti-inflammatory properties can significantly improve the likelihood of blastocyst implantation, which lowers the risk of premature delivery [40].

Polycystic ovarian syndrome

A prevalent endocrine condition known as polycystic ovary syndrome causes hormonal imbalance among females of childbearing age. The ovaries' polycystic appearance is often distinguished as visualized under ultrasonography or USG. Other features like hyperandrogenism and ovulatory dysfunction, often leading to anovulation and irregular menstrual cycles, are also observed in this condition [41]. Due to the irregularity of ovulation, conceiving a child is more difficult for women with polycystic ovarian syndrome. Moreover, these individuals are more susceptible to experiencing pregnancy-related complications [42]. The metabolic abnormalities associated with this syndrome, like insulin resistance, obesity, and hyperandrogenism, may contribute to neonatal and obstetric complications in such pregnancies. These complications include a twofold increased risk of premature delivery, a threefold increased risk of developing gestational diabetes mellitus [43], and a threefold to fourfold increase in developing pregnancy-induced hypertension (PIH) or pre-eclampsia [44]. Further complications can be attributed to the inflammation, thrombosis, and infarction of the placenta observed in females with polycystic ovary syndrome. When this is coupled with nucleated red blood cells and villous immaturity, it may indicate fetal hypoxia and vascular damage [45].

Patients with the polycystic ovarian syndrome who are not pregnant are frequently prescribed metformin. It tends to have an anti-androgenic effect and increases insulin sensitivity, ovulation, and menstrual cyclicity [46]. It is often used with clomiphene citrate to treat subfertility, particularly in clomiphene citrate-resistant women [47].

The two thiazolidinedione drugs-rosiglitazone and pioglitazone-are categorized under pregnancy category C drugs as in experiments on animals they demonstrated a reduction in fetal growth. Therefore, those pregnancies associated with polycystic ovary syndrome are mainly administered metformin because there is no proof that this medication increases the chance of gross abnormalities during pregnancy, making it a category B drug. [48,49]. Metformin usage has shown a lowered incidence of miscarriage in such patients, wherein the incidence is 18.7% in untreated patients. In contrast, for patients who had metformin medication for the first 32 weeks of pregnancy, the incidence is 10%. Meanwhile, it has even dropped to 0% if they underwent metformin therapy throughout their pregnancy [50]. Some studies have suggested that using metformin in such pregnancies is linked with a reduced incidence of androgen excess in the fetus and fewer chances of gestational diabetes mellitus developing in such pregnancies, especially when supplemented with dietary control [51]. Studies on neonatal outcomes of metformin usage in pregnant women who have polycystic ovary syndrome suggest that the drug does not exhibit teratogenic properties [52] and, therefore, does not negatively impact growth, birth weight, and motor-social development during the first one and a half years of the life of the child [53]. Under metformin therapy, especially in the preconception period, pregnancies tend to be singleton pregnancies. Moreover, the patient is less likely to develop OHSS or ovarian hyperstimulation syndrome [54].

Fetal outcomes associated with the use of metformin during pregnancy

As per the Food and Drug Administration (FDA), metformin is a category B medication, meaning animal trials using the drug have not revealed any substantial risks or negative effects for the fetus; however, the same has not been conducted in adequate and well-controlled human patients. It has been over 40 years since metformin began to be used during pregnancy. Since then, there have been multiple studies to evaluate the outcomes of such pregnancies to ensure the safe prescription of the drug.

Certain cohort studies and randomized controlled trials have concluded that neonatal hypoglycemia is significantly less common when metformin is used. Additionally, there have been fewer admissions of neonates to the NICU than insulin use [55]. A network meta-analysis of 32 randomized controlled trials revealed metformin is considered superior to hypoglycaemic drugs like insulin and glyburide in reducing respiratory distress, pregnancy-induced hypertension, macrosomia, and LGA babies, especially in obese mothers with gestational diabetes mellitus [56].

Short-term follow-up of pregnancies that used metformin therapy revealed that there was not any higher chance of pre-eclampsia, premature labor, neonatal intensive care unit admissions, macrosomia or microsomia, and morbidity or mortality of the child up to the first year of life [26]. However, long-term follow-ups of such pregnancies revealed specific noteworthy outcomes, as listed in Table 2.

**Table 2:** Outcomes of pregnancies under metformin therapy upon long-term follow up

Randomized controlled trials	Condition of the mother	Follow-up age of the child	Findings observed
Hanem et al. [57]	Polycystic ovary syndrome	Four years	Children of mothers with metformin medication during pregnancy had a higher body mass index and were more likely to become obese later in life.
Ro et al. [58]	Polycystic ovary syndrome	Seven to nine years	Children receiving metformin medication had a marginally elevated fasting blood sugar level—a possible correlation between lower LDL cholesterol levels and higher systolic blood pressure.
Rowan et al. [59]	Gestational diabetes mellitus	Seven to nine years	There is a higher than normal measurement of weight, waist and arm circumference, abdominal fat volume, triceps skinfold, and BMI in these children.
Rowan et al. [23]	Gestational diabetes mellitus	Two years	Increased subcutaneous fat was observed in these children
Ijaz et al. [60]	Gestational diabetes mellitus	Twelve months and eighteen months	Higher than normal body weight in the children exposed to metformin therapy

Due of the drug's tendency to cross the placenta readily and expose the fetus to it, the use of metformin during pregnancy is a widely contested subject. The fetus is exposed to high metformin levels a few hours after it has been administered to the mother [61]. This increased exposure to metformin may be because metformin is primarily excreted through the renal route in adults. Still, in the fetus, it is excreted into the amniotic fluid, which could be swallowed back by them, thereby re-entering their circulation [62]. Therefore, it poses a potential problem because there has not been enough research on metformin metabolism in the fetus, and thus, knowledge on the subject is scarce [61].

Generally speaking, metformin is not regarded as a teratogenic medication; therefore, healthcare professionals usually prescribe it without expecting birth defects in the fetus. However, some controlled studies have observed certain birth defects in pregnant women with polycystic ovary syndrome and diabetes mellitus who were given metformin, as seen in Table 3.

**Table 3:** Observed birth defects seen in studies done on metformin-exposed pregnant groups Cassina M, Donà M, Di Gianantonio E, Litta P, Clementi M: First-trimester exposure to metformin and risk of birth defects: a systematic review and meta-analysis. Hum Reprod Update. 2014, 20:656–69. 10.1093/humupd/dmu022 [67].

Author	Condition of the mother for which she was receiving metformin	Sample size	Methodology	Outcomes observed in the metformin-exposed groups
Coetzee and Jackson [63]	Pre-pregnancy type 2 diabetes mellitus	171 pregnant women with non-insulin-dependent diabetes	Results were compared between 78 patients who were given oral hypoglycaemic drugs during their first trimester and 93 patients who were not over 5.5 years	Twenty individuals whose mothers received Glibenclamide and metformin in the first six months of pregnancy had polydactyly.
Moll et al. [64]	Polycystic ovary syndrome	228 patients with the polycystic ovarian syndrome	In a randomized controlled trial where 111 patients were given clomiphene citrate along with metformin, and 114 were given clomiphene citrate with a placebo. The ovulation rates, the rates of ongoing pregnancy, and the abortion rates were compared between the metformin and placebo groups.	Two cases with anomalies were reported in the metformin-exposed group. One case had anal atresia (a birth defect), and the other had Kartagener's syndrome (a genetic disease).
Hughes and Rowan [65]	Pre-pregnancy type 2 diabetes mellitus	214 patients with type 2 diabetes mellitus	Metformin was given to 93 pregnancies which were continued till delivery in 32 pregnancies. 121 pregnancies were in the control group. The study took place over six years, where the data were collected by case-note review.	No congenital malformations were observed; however, one instance of tetralogy of Fallot was noted in the group that had taken metformin.
Legro et al. [66]	Polycystic ovary syndrome	626 infertile women with polycystic ovary syndrome	The candidates were distributed into three study groups – one group receiving clomiphene citrate, another extended-release metformin plus placebo, and the last, a combination of metformin and clomiphene for six months.	The metformin-exposed group reported two instances where one had a congenital diaphragmatic hernia, and the other case had Prader-Willi syndrome (a genetic disease).

A link exists between metformin usage during pregnancy and small-for-gestational age births because metformin affects the bioavailability of nutrients and fetal growth by inhibiting mitochondrial complex I, which activates AMPK signaling and inhibits placental mTOR signaling [68]. Metformin can further affect fetal and placental development along with increasing the incidence of cardiometabolic complications in the fetus by resulting in an unbalanced level of folate and vitamin B12; therefore, vitamin supplementation is recommended before the administration of metformin for a pregnancy to reduce the incidence of small-for-gestational-age babies and childhood obesity [42,62].

## Conclusions

For more than forty years now, metformin has been a routinely prescribed drug for diabetes mellitus, which has also seen its use in certain pregnancies. These pregnancies include those of obese mothers, mothers with polycystic ovarian disorder, and gestational diabetes mellitus. However, there still exists a grey area when prescribing metformin for pregnancy. Even though its benefits have been more or less established through findings from various controlled trials, one has to weigh the benefits against the risks posed by the drug, as not much research has been conducted regarding the mechanisms of its metabolism in the fetus. All the information on metformin as a drug for pregnancy comes from studies that are often underfunded, do not have enough study participants, and do not conduct long-term follow-ups. As a result of this, regarding the drug's safety, there are no readily available definite facts. The number of maternal gestational diabetes mellitus and polycystic ovary syndrome cases has only gone up over the years. Thus, it becomes increasingly important to conduct more high-quality research on fetal outcomes of metformin administration during pregnancy.

## References

[1] Nasri H, Rafieian-Kopaei M (2014). Metformin: current knowledge. J Res Med Sci.

[2] Bailey CJ, Day C (1989). Traditional plant medicines as treatments for diabetes. Diabetes Care.

[3] Pryor R, Cabreiro F (2015). Repurposing metformin: an old drug with new tricks in its binding pockets. Biochem J.

[4] Thomas I, Gregg B (2017). Metformin; a review of its history and future: from lilac to longevity. Pediatr Diabetes.

[5] Todd JN, Florez JC (2014). An update on the pharmacogenomics of metformin: progress, problems and potential. Pharmacogenomics.

[6] Jorquera G, Echiburú B, Crisosto N, Sotomayor-Zárate R, Maliqueo M, Cruz G (2020). Metformin during pregnancy: Effects on offspring development and metabolic function. Front Pharmacol.

[7] Pernicova I, Korbonits M (2014). Metformin--mode of action and clinical implications for diabetes and cancer. Nat Rev Endocrinol.

[8] He L, Wondisford FE (2015). Metformin action: concentrations matter. Cell Metab.

[9] Domecq JP, Prutsky G, Leppin A (2015). Clinical review: drugs commonly associated with weight change: a systematic review and meta-analysis. J Clin Endocrinol Metab.

[10] Motta AB (2009). Mechanisms involved in metformin action in the treatment of polycystic ovary syndrome. Curr Pharm Des.

[11] Lavine JE, Schwimmer JB, Van Natta ML (2011). Effect of vitamin E or metformin for treatment of nonalcoholic fatty liver disease in children and adolescents: the TONIC randomized controlled trial. JAMA.

[12] Nimako GK, Wintrob ZA, Sulik DA, Donato JL, Ceacareanu AC (2017). Synergistic benefit of statin and metformin in gastrointestinal malignancies. J Pharm Pract.

[13] Hyer S, Balani J, Shehata H (2018). Metformin in pregnancy: mechanisms and clinical applications. Int J Mol Sci.

[14] (2022). Hyperglycaemia in pregnancy (HIP) (20-49 y): prevalence of gestational diabetes mellitus (GDM). http://diabetesatlas.org/data/.

[15] Anthony N, Ahmad A, Bibi C (2021). Feto-maternal outcomes and treatment compliance in metformin Versus insulin-treated gestational diabetic and non-diabetic patients at the Rehman Medical Institute, Peshawar. Cureus.

[16] (2022). Gestational diabetes. http://www.idf.org/our-activities/care-prevention/gdm.

[17] Panaitescu AM, Ciobanu AM, Popa M, Duta I, Gica N, Peltecu G, Veduta A (2021). Screening for gestational diabetes during the COVID-19 pandemic-current recommendations and their consequences. Medicina (Kaunas).

[18] Denison FC, Aedla NR, Keag O, Hor K, Reynolds RM, Milne A, Diamond A (2019). Care of women with obesity in pregnancy: Green-top Guideline No. 72. BJOG.

[19] (2022). Management of diabetes. https://www.sign.ac.uk/assets/sign116.pdf.

[20] Kim C, Ferrara A (2010). Gestational Diabetes During and After Pregnancy.

[21] (2020). Management of diabetes in pregnancy: standards of medical care in diabetes-2020. Diabetes Care.

[22] (2022). Diabetes in pregnancy: management from preconception to the postnatal period. https://www.nice.org.uk/guidance/ng3.

[23] Rowan JA, Hague WM, Gao W, Battin MR, Moore MP (2008). Metformin versus insulin for the treatment of gestational diabetes. N Engl J Med.

[24] Gui J, Liu Q, Feng L (2013). Metformin vs insulin in the management of gestational diabetes: a meta-analysis. PLoS One.

[25] Farrar D, Simmonds M, Bryant M, Sheldon TA, Tuffnell D, Golder S, Lawlor DA (2017). Treatments for gestational diabetes: a systematic review and meta-analysis. BMJ Open.

[26] Butalia S, Gutierrez L, Lodha A, Aitken E, Zakariasen A, Donovan L (2017). Short- and long-term outcomes of metformin compared with insulin alone in pregnancy: a systematic review and meta-analysis. Diabet Med.

[27] (2022). Obesity and overweight. https://www.who.int/news-room/fact-sheets/detail/obesity-and-overweight.

[28] Guelinckx I, Devlieger R, Beckers K, Vansant G (2008). Maternal obesity: pregnancy complications, gestational weight gain and nutrition. Obes Rev.

[29] Braeken MA, Bogaerts A (2020). Effect of lifestyle interventions in obese pregnant women on the neurocognitive development and anthropometrics of preschool children. Obes Facts.

[30] Sutherland HW, Stowers JM (1984). Carbohydrate Metabolism in Pregnancy and the Newborn. https://link.springer.com/book/10.1007/978-1-4471-1680-6.

[31] Ramsay JE, Greer I, Sattar N (2006). ABC of obesity. Obesity and reproduction. BMJ.

[32] Sebire NJ, Jolly M, Harris JP (2001). Maternal obesity and pregnancy outcome: a study of 287,213 pregnancies in London. Int J Obes Relat Metab Disord.

[33] Baeten JM, Bukusi EA, Lambe M (2001). Pregnancy complications and outcomes among overweight and obese nulliparous women. Am J Public Health.

[34] Kristensen J, Vestergaard M, Wisborg K, Kesmodel U, Secher NJ (2005). Pre-pregnancy weight and the risk of stillbirth and neonatal death. BJOG.

[35] Cedergren MI (2004). Maternal morbid obesity and the risk of adverse pregnancy outcome. Obstet Gynecol.

[36] Mahmood T, Arulkumaran S, Chervenak F (2020). Obesity and Obstetrics. Elsevier.

[37] Watkins ML, Rasmussen SA, Honein MA, Botto LD, Moore CA (2003). Maternal obesity and risk for birth defects. Pediatrics.

[38] D'Ambrosio V, Brunelli R, Vena F (2019). Metformin reduces maternal weight gain in obese pregnant women: a systematic review and meta-analysis of two randomized controlled trials. Diabetes Metab Res Rev.

[39] Syngelaki A, Nicolaides KH, Balani J (2016). Metformin versus placebo in obese pregnant women without diabetes mellitus. N Engl J Med.

[40] Sun X, Tavenier A, Deng W, Leishman E, Bradshaw HB, Dey SK (2018). Metformin attenuates susceptibility to inflammation-induced preterm birth in mice with higher endocannabinoid levels. Biol Reprod.

[41] Deans R (2019). Polycystic ovary syndrome in adolescence. Med Sci (Basel).

[42] Maxwell C, Farine D (2017). Pregnancy and Obesity. De Gruyter.

[43] Dunning AM T Care of People with Diabetes: A Manual of Nursing Practice. 1st ed.

[44] Palomba S, de Wilde MA, Falbo A, Koster MP, La Sala GB, Fauser BC (2015). Pregnancy complications in women with polycystic ovary syndrome. Hum Reprod Update.

[45] Koster MP, de Wilde MA, Veltman-Verhulst SM, Houben ML, Nikkels PG, van Rijn BB, Fauser BC (2015). Placental characteristics in women with polycystic ovary syndrome. Hum Reprod.

[46] Nestler JE (2008). Metformin for the treatment of the polycystic ovary syndrome. N Engl J Med.

[47] Creanga AA, Bradley HM, McCormick C, Witkop CT (2008). Use of metformin in polycystic ovary syndrome: a meta-analysis. Obstet Gynecol.

[48] Petitti DB (2003). Clinical practice. Combination estrogen-progestin oral contraceptives. N Engl J Med.

[49] Gilbert C, Valois M, Koren G (2006). Pregnancy outcome after first-trimester exposure to metformin: a meta-analysis. Fertil Steril.

[50] Nawaz FH, Khalid R, Naru T, Rizvi J (2008). Does continuous use of metformin throughout pregnancy improve pregnancy outcomes in women with polycystic ovarian syndrome?. J Obstet Gynaecol Res.

[51] Glueck CJ, Goldenberg N, Wang P, Loftspring M, Sherman A (2004). Metformin during pregnancy reduces insulin, insulin resistance, insulin secretion, weight, testosterone and development of gestational diabetes: prospective longitudinal assessment of women with polycystic ovary syndrome from preconception throughout pregnancy. Hum Reprod.

[52] Farid NR, Diamanti-Kandarakis E (2009). Diagnosis and Management of Polycystic Ovary Syndrome.

[53] Hellmuth E, Damm P, Mølsted-Pedersen L (2000). Oral hypoglycaemic agents in 118 diabetic pregnancies. Diabet Med.

[54] Mathur R, Alexander CJ, Yano J, Trivax B, Azziz R (2008). Use of metformin in polycystic ovary syndrome. Am J Obstet Gynecol.

[55] Priya G, Kalra S (2018). Metformin in the management of diabetes during pregnancy and lactation. Drugs Context.

[56] Liang HL, Ma SJ, Xiao YN, Tan HZ (2017). Comparative efficacy and safety of oral antidiabetic drugs and insulin in treating gestational diabetes mellitus: An updated PRISMA-compliant network meta-analysis. Medicine (Baltimore).

[57] Hanem LG, Stridsklev S, Júlíusson PB (2018). Metformin Use in PCOS pregnancies increases the risk of offspring overweight at 4 years of age: follow-up of two RCTs. J Clin Endocrinol Metab.

[58] Rø TB, Ludvigsen HV, Carlsen SM, Vanky E (2012). Growth, body composition and metabolic profile of 8-year-old children exposed to metformin in utero. Scand J Clin Lab Invest.

[59] Rowan JA, Rush EC, Obolonkin V, Battin M, Wouldes T, Hague WM (2011). Metformin in gestational diabetes: the offspring follow-up (MiG TOFU): body composition at 2 years of age. Diabetes Care.

[60] Ijäs H, Vääräsmäki M, Saarela T, Keravuo R, Raudaskoski T (2015). A follow-up of a randomised study of metformin and insulin in gestational diabetes mellitus: growth and development of the children at the age of 18 months. BJOG.

[61] Vanky E, Zahlsen K, Spigset O, Carlsen SM (2005). Placental passage of metformin in women with polycystic ovary syndrome. Fertil Steril.

[62] Stowers JM, Sutherland HW (1984). The use of sulphonylureas biguanides and insulin in pregnancy. Carbohydrate Metabolism in Pregnancy and the Newborn.

[63] Coetzee EJ, Jackson WP (1984). Oral hypoglycaemics in the first trimester and fetal outcome. S Afr Med J.

[64] Moll E, Bossuyt PM, Korevaar JC, Lambalk CB, van der Veen F (2006). Effect of clomifene citrate plus metformin and clomifene citrate plus placebo on induction of ovulation in women with newly diagnosed polycystic ovary syndrome: randomised double blind clinical trial. BMJ.

[65] Hughes RC, Rowan JA (2006). Pregnancy in women with type 2 diabetes: who takes metformin and what is the outcome?. Diabet Med.

[66] Legro RS, Barnhart HX, Schlaff WD (2007). Clomiphene, metformin, or both for infertility in the polycystic ovary syndrome. N Engl J Med.

[67] Cassina M, Donà M, Di Gianantonio E, Litta P, Clementi M (2014). First-trimester exposure to metformin and risk of birth defects: a systematic review and meta-analysis. Hum Reprod Update.

[68] Grace MR, Dotters-Katz SK, Zhou C, Manuck T, Boggess K, Bae-Jump V (2019). Effect of a high-fat diet and metformin on placental mTOR signaling in mice. AJP Rep.

